# Screen Time Exposure and Domain-Specific Working Memory in Young Adults

**DOI:** 10.7759/cureus.60626

**Published:** 2024-05-19

**Authors:** Darshan H Sarvajna, Jim S Winston, Devika P S, Mariyam Nuza, Vismaya Venugopalan

**Affiliations:** 1 Nitte Institute of Speech and Hearing, Nitte Deemed to be University, Mangaluru, IND

**Keywords:** corsi backward span, reverse digit span, short term memory, working memory, screen time

## Abstract

Introduction

With technology advancing across all fields, the utility of digital screens is increasing among all age groups for various purposes. Research indicates that while digital technology presents clear advantages, prolonged exposure can have detrimental effects on various aspects of health, behavior, emotions, and cognitive functions like attention and working memory. A crucial cognitive process for learning and information processing which is working memory, can be affected by factors including screen time. Studies have found that the impact of screen time on working memory can be negative, positive, or show no discernible relationship. However, earlier investigations are limited to smartphone use as screen time exposure and further to only active screen time. As there is a dearth of studies in the Indian context and young adults are more exposed to screen time, it is important to investigate along these lines. Hence, the present study aimed to investigate the impact of active and passive screen time exposure on modality-specific working memory in young adults.

Methods

Seventy-seven neurotypical individuals aged between 18 and 22 years were recruited. The study utilized auditory and visual reverse digit span tasks and the Corsi-backward task to measure working memory span. Screen time data of the participants were collected through a self-administered 18-item questionnaire covering active and background screen time domains.

Results and discussion

The present study concluded that only active screen time has a significant effect on visual reverse digit span and supports the notion of the visual superiority effect against an auditory superior effect as suggested by earlier findings. The preliminary findings of correlation observed exclusively within the visual domain in this study could be attributed to the potential impact of screen time exposure (active screen time and textual content). Screen usage demands effective switching between various visual stimuli and ongoing updates of information in memory. Nonetheless, interpreting this explanation and generalization requires caution, given the low ecological validity of the task employed in the study. Future investigations should aim to collect screen time exposure data more objectively, perhaps through online tracking techniques. Furthermore, it would be prudent to expand the correlation analysis to include other cognitive processes and populations.

## Introduction

The widespread usage of digital gadgets, such as smartphones, tablets, and computers, has recently become a crucial aspect of daily life for individuals of all ages. Due to the rapid improvement of technology and the expanding accessibility of digital media, they are engaging with screens for extended periods for various purposes, from entertainment to work, education, and learning, all due to the rapid improvement of technology and the expanding accessibility of digital media. Despite the apparent advantages of digital technology, research has proven that intensive exposure can affect general health, behavioral, and emotional problems [1], and cognitive processes like attention, working memory (WM), and many others across age groups [2,3].

Working memory is crucial in various cognitive processes, including attention, problem-solving, decision-making, and learning. It is a cognitive process that temporarily stores and manipulates information needed for ongoing mental tasks [4]. Working memory performance is unstable volatile and sensitive to many social and situational factors, such as stress, sleep, and certain mental illnesses [5]. Another factor recently gaining focus is screen time, which is among the mentioned factors, screen time, is used for activities like learning, updating, and recalling information across various sensory domains in the current trends. Given the essential nature of working memory and the effects of screen time on everyday functioning, any factors that may influence working memory performance warrant a thorough investigation.

A review of available reports on the essential of investigating the correlation between the influence of screen time and working memory reveals an unequivocal juncture in scientific knowledge. There are four primary schools of thought; the first suggests that screen time adversely affects working memory [6,7]. A few other reports provide evidence of a beneficial impact on working memory domains [3]. The third school of reports points towards a neutral impact of screen time on tasks tapping working memory [8]. Some studies have suggested that excessive screen time may negatively impact working memory. Baumgartner et al. [9] found no relationship between media multitasking and working memory, where working memory was assessed using digit-span tasks. However, Cain et al. [10] investigated the impact of media multitasking on working memory, verbal comprehension, calculation, cognitive processing speed, manual dexterity, and implicit learning. The working memory span was indexed using the n-back task. In this study, the researchers found a negative correlation between media multitasking and working memory i.e., greater media multitasking related to a reduction in the executive function component of working memory. Similarly, Abramson et al. [6] found higher mobile phone usage was associated with decreased accuracy in working memory and associative learning tasks, along with quicker reaction times on simple and associative learning tasks in 13-year-old school-going children. This implies that rather than affecting just one cognitive function, the use of mobile phones might be tied to an impulsive response pattern in children. Soares et al. [8] provided further evidence supporting these findings. They studied 3,625 teenagers at various age stages, examining the impact of three types of screen time-watching television, playing video games, and using computers-on their working memory, using the reverse digit span test. The study revealed a complex relationship between screen time and working memory, with some positive, negative, and non-significant outcomes. When looking into television viewing, computer use, and video game usage between ages 11 and 15 showed a strong correlation with improved backward digit span performance significantly in males compared to females.

Another group of researchers has proposed that the relationship between screen time and working memory might be more complex, potentially influenced by content/stimulus type, screen context, and individual differences [11,12]. The limited sparsity of research on the correlation between influence of screen time and working memory in young adults, coupled with the inconclusive findings, underscores the necessity for further exploration of this relationship.

Most of these studies in this line have focused on considering smartphone usage as screen time [11,12], thereby failing to account for the broader spectrum of overall screen time, encompassing smartphone use, laptop use, and television viewing, among other activities. Given the diverse range of activities associated with screen time, both in terms of type and quantity duration, this comprehensive screen time data is crucial to establish correlations with cognitive processes given the inconsistent findings found in the literature. Furthermore, it is important essential to distinguish between active screen time (AST) and background screen time (BST) on these lines. This differentiation becomes essential when quantifying the potential effects or correlation with the working memory performance. AST is the time spent directly interacting with the screen as the primary activity. BST use comprises using a screen as a secondary media in the vicinity while engaging in other activities such as cooking, eating or exercising. 

As mentioned earlier, working memory is a critical cognitive process, which is highly susceptible to positive and negative influences from the experiences gained. Therefore, it is paramount to assess working memory across visual, auditory, and visuo-spatial domains while correlating it with an individual's exposure to screen time particularly in young adults considering significant use of screen time for various purposes in their past years. An additional vital aspect of the current study is the dearth of studies addressing these specific lines of work in an Indian context, prompting the researchers to design and investigate the correlation between active and background screen time exposure and domain-specific working memory in young adults. The specific objectives of the current study are a) to collect screen time exposure information; b) To assess working memory across domains in young adults; and c) to assess the correlation between working memory and screen time exposure.

## Materials and methods

Participants

Utilizing the convenience sampling method, 77 participants, ranging in age from 18 to 22 years (Males=20.32, SD=1.6, Females=34), were recruited for the study. All the participants were Malayalam-English bilingual undergraduate university students from the Dakshina Kannada District of Karnataka, India. The participants were invited to take part in the study by one of the researchers, who explained the details of the experiment to them face-to-face. Participation in the study was voluntary, and written informed consent was obtained from all the participants in compliance with the Declaration of Helsinki [13]. 

Self-reported data from all participants indicated a good quality of life in the physical, psychological, social relationships, and environment domains of the World Health Organization Quality of Life Brief Version (WHOQOL-BREF) [14]. The pure-tone audiometry thresholds at standard audiometric test frequencies of 250 to 8000Hz were within 15dBHL for air conduction, suggesting normal hearing sensitivity [15]. Pass in the Screening Checklist for Auditory Processing Disorders in Adults (SCAP-A) ruled out auditory processing deficits [16]. The six-meter standard Snellen visual acuity test was used to ensure a 6/6 score in visual acuity of all participants with or without correction [17]. No other inclusion or exclusion criteria were used.

Procedure

Screen Time Questionnaire

Participants' screen time data was collected using a self-administered 18-item questionnaire in the domains of AST and BST [18]. The questionnaire prompted participants to give information about the total duration spent in hours and minutes on activities involving TV, TV-connected devices (such as media streaming devices and gaming consoles), laptops/computers, smartphones, and tablets. AST is the time spent directly interacting with the screen as the primary activity. BST use comprises using a screen as a secondary media in the vicinity while engaging in other activities such as cooking or exercising.

Auditory Reverse Digit Span

The Auditory Reverse Digit Span (ARDS) task measured working memory in the auditory domain. The experiment was designed using Paradigm Stimulus Presentation Software [19] and was run on a Dell Inspiron 15 3000 series laptop (Dell Inc. Round Rock, TX, USA) using Paradigm Player. English single-digit numbers from one to nine spoken by a female speaker were played back to the participants through Audio-Technica ATH-M50X headphones (Audio-Technica Corporation, Tokyo, Japan). Each trial of the experiment consisted of a string of digits varying in length from three to eight in increasing order of complexity, and five trials were provided for each string length. The inter-stimulus interval between two consecutive digits was 500 ms, and the inter-trial interval was 10s, including response time. Before each trial, a black '+' sign appeared at the center of the screen on a white background for 500 ms. The participants were instructed to listen carefully to the digits and respond by typing them on the keyboard in reverse order at the end of each trial.

Visual Reverse Digit Span

A Visual Reverse Digit Span (VRDS) task was used to measure the working memory in the visual domain. The design and execution of the experiment were similar to the ARDS experiment used in the present study. However, the digits were presented in the visual modality using a laptop screen of diagonal length 15.6 inches placed at 0-degree azimuth and at a distance of 75 cm in a room free from noise and visual distractors. The digits appeared at the center of the screen for a duration of 500ms and measured 1 inch in dimension. Similar to the ARDS task, a fixation '+' appeared on the screen, marking each new trial's beginning. At the end of each trial, the participants recalled the string of digits in reverse order and typed the response on the keyboard.

Backward Corsi Task

The Backward Corsi task (CB) measured the working memory span in the visuospatial domain using PsychToolkit [20]. The pre-downloaded experiment was run using Google Chrome on a display setup similar to the VRDS task. In the CB task, highlighted squares measuring one inch appeared randomly and sequentially within a larger square measuring 8 inches wide and 6 inches in height. The number of squares varied as a function of complexity from three to eight. Other parameters used were similar to the VRDS and ARDS tasks. At the end of each trial, the participants responded using the mouse by clicking the rectangles starting from the last highlighted to the first highlighted. 

**Figure 1:**
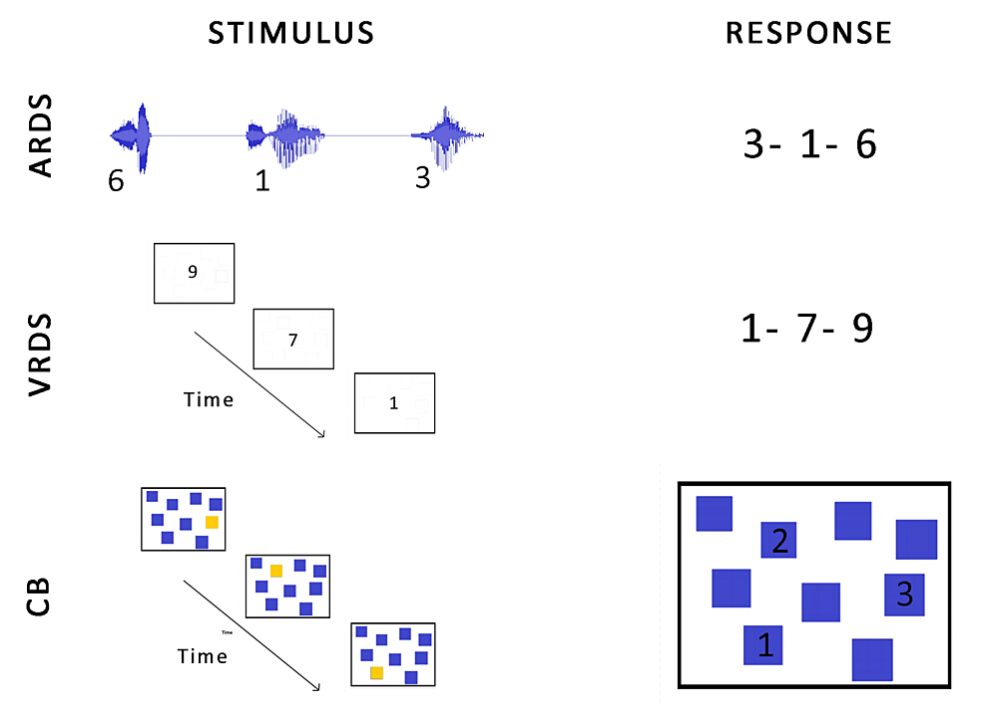
The stimulus delivery, and expected response for auditory reverse digit span (ARDS), visual reverse digit span (VRDS) and backward Corsi (CB) tasks used in the present study.

The illustration of three WM tasks is represented in Figure 1, which provides information about stimulus delivery and expected responses.

Scoring

For all the working memory tasks, the minimum string level at which the participants consistently recorded a minimum of three correct responses out of five trials was considered the working memory span for the particular measure. In simple words, working memory span refers to the length of the longest string the participant recalls in reverse order more than 60% of the time. The output file from the Paradigm software for WM tasks was manually verified and noted WM span by the third and fourth researchers of the study.

## Results

The present study explored the impact of screen time on ARDS, VRDS, and CB in young adults. The data gathered were subjected to statistical analysis using JASP (0.16.1) [21]. The significance level was set at alpha < 0.05.

Screentime characteristics

The mean screen times for the AST and BST groups were 18.49±6.61 hours and 4.33±3.58 hours, respectively. The participants were divided into high and low screen time groups based on the 50th percentile value of the AST (18 hours) and BST (four hours) for further comparisons.

**Figure 2:**
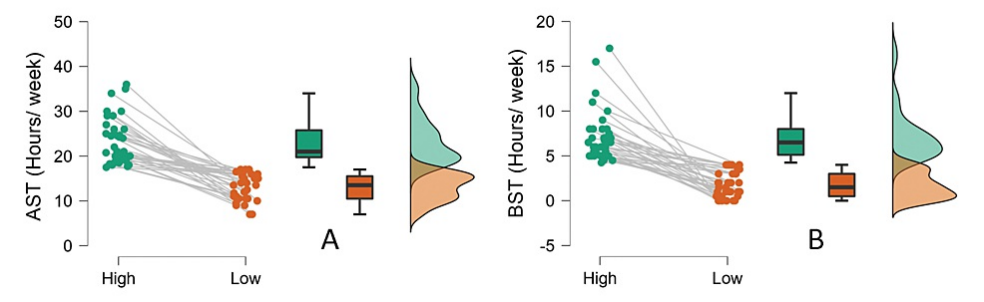
(A) Active screen time (AST) and (B) background screen time (BST) in the high and low screen time groups

The Shapiro-Wilk test was used to test data compliance with the normality assumption. The results revealed normal distribution only for the low active screen time group (*p*=0.06) and not for the other subgroups (*p*<0.05). Further, the Wilcoxon signed-rank test was used to compare the hours/week across low and high screen time users in the AST and BST groups. The results revealed that the high screen time group had significantly higher screen time usage compared to the low screen time users in the AST (Z=5.01, *p*<0.001, rrb=1.0) (Figure 2A) and BST group (Z=5.08, *p*<0.001, rrb=1.0) (Figure 2B).

Screentime and working memory

**Figure 3:**
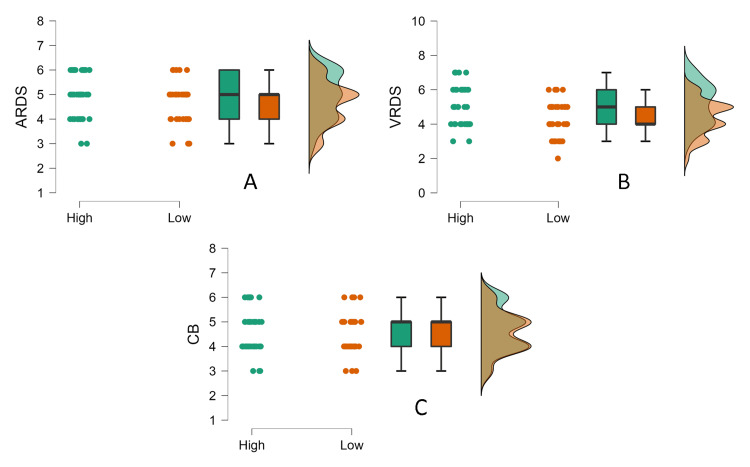
(A) Auditory reverse digit span (ARDS), (B) visual reverse digit span (VRDS), and (C) Corsi-backward (CB) span in the high and low active screen time (AST) groups

Since the data violated the normality assumption (p<0.05), the Mann-Whitney test was used to compare the effect of screen time on ARDS, VRDS, and CB. The results revealed no significant impact of low vs. high AST on ARDS (U=751.50, p=.134, rrb=0.199) (Figure 3A). However, the high AST group performed significantly better than the low AST group in the VRDS task (U=850, p=.008, rrb=0.356) (Figure 3B). Further, no significant difference in performance was present in the CB task (U=678, p=.537, rrb=0.081) (Figure 3C). BST was revealed to have no significant effect on ARDS (U=553, p=.362, rrb= -0.121), VRDS (U=504, p=.139, rrb=-0.199), or CB (U=507.5, p=.140, rrb= -0.193). BST was revealed to have no significant effect on ARDS (U=553, *p*=.362, rrb= -0.121), VRDS (U=504, *p*=.139, rrb=-0.199), or CB (U=507.5, *p*=.140, rrb= -0.193).

Further, the Friedman test was used to compare the performances of each AST and BST sub-groups across ARDS, VRDS, and CB tasks. The results revealed no significant group effect in the high AST (χ2(2) = 4.00, *p* = 0.135), low AST (χ2(2) = 0.485, *p* = 0.135), high BST (χ2(2) = 1.978, *p* = 0.372), or low BST groups (χ2(2) = 0.569, *p* = 0.752).

**Figure 4:**
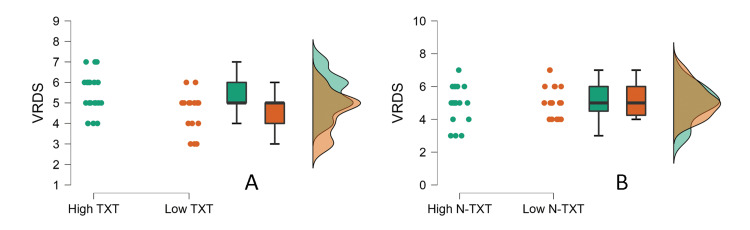
Visual reverse digit span (VRDS) in the high and low (A) textual (TXT) and (B) non-textual (N-TXT) groups.

Since the analysis revealed a significant effect of AST on VRDS scores, it was interesting to explore whether the content viewed by the high AST group influenced the VRDS score. Hence, the participants in the high AST were contacted retrospectively and asked to answer a single question on how many hours of the AST they reported were spent on textual content. The difference between the reported AST and textual hours was taken as non-textual. The 50th percentile of textual (eight hours) and non-textual (14.6 hours) was used to divide the high AST group into low and high textual/non-textual groups. The VRDS scores of the high and low textual and high and low non-textual groups were compared using the Mann-Whitney U test. The results revealed that the high textual group performed significantly better than the low textual group (U=237.0, *p*=0.021, rrb=0.436) (Figure 4A), and no difference was observed in the non-textual group (U=160.00, *p*=0.73, rrb=-0.060) (Figure 4B).

Correlation between screentime and working memory

**Figure 5:**
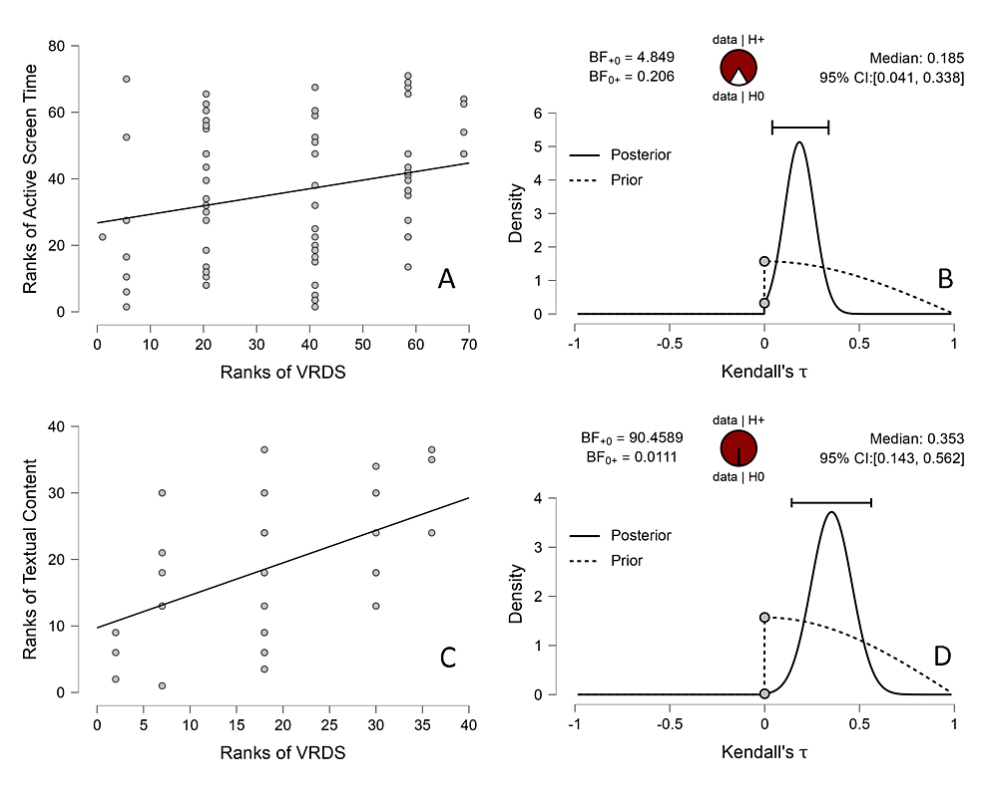
Correlation matrix (left side) and posterior-prior plots (right side) resulting from the one-tailed Bayesian Kendall's Tau correlation between visual reverse digit span (VRDS) and (A, B) active screen time and (C, D) textual content.

Kendall's Tau correlation revealed a significant positive correlation only between AST and VRDS scores (τb=0.192, *p*=.03) but not for ARDS (τb=0.116, *p*=.20) or CB (τb=0.053, *p*=.56). No significant correlation was observed between BST and ARDS (τb=-0.121, *p*=.19), VRDS (τb=-0.126, *p*=.16) or CB (τb=-0.063, *p*=.49). Further, one-tailed Bayesian Kendall's tau correlation was administered to test the probability of the data aligning with the alternative hypothesis (H1) that AST and VRDS scores are positively correlated against H0. The resulting BF+0 is 4.85 (τb= 0.192), indicating moderate evidence favoring H1, implying that the data is 4.8 times more likely to occur under H1 than under H0 (Figure 5A, 5B).

A positive correlation was observed between VRDS and hours of textual content (τb=0.382, *p*=.004) but not with non-textual content (τb=-0.071, *p*=.58). One-tailed Bayesian Kendall's tau correlation revealed very strong evidence (BF+0=90.45, τb=0.382) in favor of the alternating hypothesis postulating a positive correlation between VRDS and hours of textual content against H0 (Figure 5C, 5D).

## Discussion

The study investigated the working memory span measured in visual and auditory domains and its correlation with active and passive screen time. In the current study, using digit span tasks to assess working memory can be advantageous, considering the uniformity in the stimulus across participants and its overlearned nature, which could be helpful in assessing core working memory capacity. The results revealed a significant influence of high AST on VRDS but no substantial impact on ARDS and CB span tasks. This finding contradicts the conclusions of earlier research [8,9,22], reports on modality-non-specific memory [23], and challenges the notion of the auditory superiority effect [24]. However, the present findings align with reports on visual superiority in the early stages of the working memory process [25]. The noticeable difference in performance observed in the high AST group in the current study aligns with earlier reports [10,26]. The former study found that media multitasking does not hinder attentional control. Instead, it enhances the ability to efficiently switch tasks when necessary, as demonstrated in task-switch trials [26]. The latter study, which involved a longitudinal research design, observed the relationship between higher television, computer, and video-game playing screen time at the age of 11-15 years and better working memory performance at 22 years in the men's group over women.

In the present study, when the AST group was stratified based on high and low textual content and non-textual content hours, the results revealed a better VRDS span among individuals with high textual content exposure, such as screen time. It can be postulated that a correlation exists between the AST high textual group and enhanced working memory span owing to their exposure to textual content compared to non-textual content. Moreover, participants get independence in encoding digit sequences in their preferred language during visual presentations instead of auditory ones [22]. This autonomy may have contributed to participants performing relatively better than in the ARDS task. However, this assumption warrants caution because digit sequence encoding could occur in their preferred language, but their response strategy involved typing digits on an English keyboard. Nevertheless, this approach might have facilitated participants in retaining visually presented stimuli in memory with the readily available digit representation on the keyboard.

On the other hand, it is worth noting that researchers in the literature have contended that auditory presentation tends to result in more reliable phonological code compared to visual presentation of digits. This assertion is based on the premise that in the visual task, subjects must access and retrieve the word names of digits, whereas, in the auditory task, the word names of digits are presented [27]. This potential limitation should be addressed with suitable methodological variation in future research to elucidate the superiority of visual working memory task performance.

Given this insight, it can be postulated that the preliminary findings of correlation observed exclusively within the visual domain of the current study might be attributed to the potential impact of screen time exposure (active screen time and textual content). Further, screen usage necessitates efficient switching between multiple contents in the visual domain and requires constant information holding in memory while updating it with the oncoming stream of new information [28]. However, it is vital to approach this explanation with caution due to the nature of the visual digit-span task, which enables participants to encode digit sequences in their preferred language, aiding later recall.

The understanding of the differential impact of screen time exposure (AST) and visual working memory in the present study coupling with the earlier research findings of digital media consumption and its impact on cognition i.e., working memory, attention, enhanced visual processing, and speed of processing specifically in gamers [29], could imply that the use of screen time, especially involving textual content, could draw attention to recommendations regarding screen usage. However, while acknowledging the impact of screen exposure on cognition, the authors caution that manipulating this variable poses difficulties, resulting in the study's findings being described as correlational rather than causal. Establishing the causal direction of this relationship will necessitate more robust training methods or longitudinal designs. Hence, the authors of the study are cautioned against making recommendations based solely on the findings of the study, as there is a greater scope to control variables, gather objective data about screen time exposure, and analyze cognitive processes with objective quantification.

The present study bears its limitations. Firstly, the use of convenience sampling methods to recruit participants to the study limits external validity. Secondly, the screen time levels of participants relied on subjective self-reported data, which could introduce biases. Therefore, future investigations should endeavor to gather this data more objectively, potentially through online tracking methods. In addition, the study exclusively concentrated on the working memory process, overlooking other cognitive functions. Subsequent research could employ diverse forms of WM tasks to augment the reliability of the current study's results. Furthermore, broadening the study's scope to encompass other age demographics and differences in performance between males and females would yield a more comprehensive understanding of the association between screen time and WM span.

## Conclusions

The present study delved into exploring the correlation between WM span, assessed across domains using the reverse digit span task and the CB task, and screen time exposure. The findings unveiled that active screen time levels solely correlated with WM span measured in the visual reverse digit span task. Notably, participants with higher exposure to textual content exhibited superior visual WM span revealing a domain-specific transfer of abilities. While the exact underlying mechanisms of the domain-specific enhancement observed in the present study needs thorough investigation, the findings have widespread implications in the therapeutic and educational sectors. However, any such interpretation or extrapolation of the findings should be with caution and due consideration to the limitations of the present study including the cross-sectional nature of the research design and small sample size. Overall, the findings contribute to the growing body of literature on the differential impacts of screen time on cognitive functions like working memory, highlighting the need for further investigation into domain-specific effects and underlying mechanisms.
